# GPU-Accelerated PD-IPM for Real-Time Model Predictive Control in Integrated Missile Guidance and Control Systems

**DOI:** 10.3390/s22124512

**Published:** 2022-06-14

**Authors:** Sanghyeon Lee, Heoncheol Lee, Yunyoung Kim, Jaehyun Kim, Wonseok Choi

**Affiliations:** 1 Research Institute of Manufacturing and Productivity, Kumoh National Institute of Technology, Gumi 39177, Gyeongbuk, Korea; freeg159@kumoh.ac.kr; 2 Department of IT Convergence Engineering, Kumoh National Institute of Technology, Gumi 39177, Gyeongbuk, Korea; 3 Precision Guided Munition R&D Laboratory, LIGNEX1, Seongnam 13488, Gyeonggi, Korea; yunyoung.kim@lignex1.com (Y.K.); jaehyun.kim@lignex1.com (J.K.); wonseok.choi@lignex1.com (W.C.)

**Keywords:** graphics processing unit, primal-dual interior point method, model predictive control, real-time systems, integrated missile guidance and control

## Abstract

This paper addresses the problem of real-time model predictive control (MPC) in the integrated guidance and control (IGC) of missile systems. When the primal-dual interior point method (PD-IPM), which is a convex optimization method, is used as an optimization solution for the MPC, the real-time performance of PD-IPM degenerates due to the elevated computation time in checking the Karush–Kuhn–Tucker (KKT) conditions in PD-IPM. This paper proposes a graphics processing unit (GPU)-based method to parallelize and accelerate PD-IPM for real-time MPC. The real-time performance of the proposed method was tested and analyzed on a widely-used embedded system. The comparison results with the conventional PD-IPM and other methods showed that the proposed method improved the real-time performance by reducing the computation time significantly.

## 1. Introduction

When evaluating the performance of a missile system, its guidance and control system is the main factor to be considered. Traditional guidance laws, such as proportional navigation guidance law, generate acceleration commands under the given target-missile kinematics. The commands are followed by an autopilot system that generates actuator commands to achieve the desired acceleration. In general, guidance and control are designed separately without considering interactions between the two systems. Although a separated design principle has proven to be reliable and effective over the decades, the method shows degradation in combined response compared to the separated conditions. This is because traditional guidance laws cannot guarantee optimal characteristics under autopilot lags and dynamic constraints. In [1], for example, the circular navigation guidance law that theoretically promises zero miss-distance was proposed. Although their method showed a robust performance even under uncertain autopilot models, its performance has been inevitably constrained by autopilot and dynamic response.

Integrated guidance and control (IGC) design concept has been considered as an alternative to solve the afore-mentioned issues. An IGC design is a single system that performs the role of both guidance and control, generating fin commands based on missile and target states. Since control commands are designed considering the interaction between guidance and control loop, IGC has the potential to enhance missile performance. Additionally, it is helpful to reduce the number of iterations and costs for the entire design process. To perform IGC, various control techniques are introduced. From classical control techniques to various nonlinear control techniques, including feedback linearization [2], sliding mode control [3,4], backstepping control [5], dynamic surface technique [6,7,8], and optimization-based methods [9] have been applied to solve the problem. Shima et al. [3] proposed a sliding mode control (SMC)-based IGC, enhancing the robustness of overall systems. Shtessel and Tournes [4] extended the research to a higher-order SMC to attenuate the chattering problem for dual control missiles. In [6], Hou and Duan utilized dynamic surface control (DSC) for the IGC problem under unmatched uncertainties. The DSC-based IGC technique is expanded by Liang et al. [7], with the additional consideration of input saturation. Kim et al. [9] proposed an explicit solution of finite time-varying state feedback control with a feedforward term.

Of many optimization-based methods, the Model Predictive Control (MPC) technique has been thought to be a powerful solution for IGC [10,11,12,13,14,15]. Compared to the other control techniques utilized in the previous research [2,3,4,5,6,7,8,9], online MPC provides the optimal solution within certain state constraints. MPC produces a control input that minimizes the objective function specified on the receding prediction horizon. The technique repeatedly solves the finite horizon open-loop optimal control problem and implements it in the form of closed-loop control. It can be applied not only to linear time-invariant systems, but also to multivariable, time-varying nonlinear systems [16]. In general, the optimal control problem is a quadratic programming (QP) problem. As it is an explicit solution to the time-varying state feedback form, it is easy to be adopted on-board. Additionally, MPC has the advantage of being able to set state and output constraints. Especially for the missile terminal guidance phase, the acceleration limit and seeker field-of-view (FOV) are crucial constraints caused by the finite maneuver capacity and seeker’s image plane. It is essential to consider these limits as inequality constraints in the optimization problem. Considering the advantages, MPC is a suitable control technique for terminal homing guidance. 

Despite its outstanding performance, applications of online MPC have been limited to slow dynamic systems because of computational bottlenecks. The issue is mainly caused by the optimization process that requires excessive computational capacities. To ease the problem, various studies on optimization algorithms and acceleration methods are conducted. In particular, convex optimization algorithms have been considered as a conductive solution for their computational efficiency and parallelizable characteristic. Gradient-based convex optimization techniques, such as the alternating direction method of multipliers (ADMM) [17,18,19], primal-dual interior point method (PD-IPM), parallel quadratic programming (PQP) [20,21], and active set method (ASM) [22,23,24], are employed. In this work, PD-IPM [24,25], which is the most commonly used technique for convex optimization, is applied. PD-IPM is developed using the Newton direction of the optimality conditions for the logarithmic barrier problem. The method simultaneously updates primal and dual variables by setting a residual function. Compared to ASM and PQP, PD-IPM requires a smaller number of iterations to reach the desired convergence level [26,27]. Additionally, the PD-IPM technique satisfies strict interior point feasibility by adopting a backtracking line search. This eases the constraint that the initial point must be feasible. 

However, despite the high efficiency of PD-IPM, MPC for the IGC problem needs further improvements for real-time implementation. As the dynamics of the missile and target show fast responses, the update rate-of-control command should be large enough for stability and to yield a smaller miss distance [28]. Furthermore, the large size of the prediction horizon is required for precise interception performance. Consequentially, the optimization process in MPC for IGC demands frequent operations of multiplication and inversion for large-sized matrix. For this reason, we adopt the parallel design for real-time GPU implementation. Research on accelerating the PD-IPM is conducted, as shown in Table 1. Even though there is limited research [29,30,31,32,33,34,35,36] that deals with the real-time problem of PD-IPM, it focuses on the acceleration of the linear equation solver part of PD-IPM. However, except for the linear equation solver part, we found that the KKT condition construction part also requires considerable computation time. Moreover, there is no related work that applies the PD-IPM to IGC systems. 

In this paper, we propose a GPU-accelerated PD-IPM method, which is conducted in MPC for real-time IGC systems, which parallelizes the KKT condition construction part to reduce the computation time of the PD-IPM. A series of complex matrix operations are performed on the KKT condition construction. The proposed method transforms these complex matrix operations into easier forms in the context of parallelization. Then, the transformed matrices are reformed to sparse matrices. Finally, parallelization is conducted with the sparse matrices through both built-in and customized CUDA kernels. The contributions of this paper are as follows.

- This is the first approach to accelerate missile MPC on GPU.
- The problem of considerable computation time in the KKT condition construction part of PD-IPM is firstly addressed and analyzed.
- A new parallelization method is developed for the KKT condition construction part of PD-IPM.
- The computation time for PD-IPM is significantly reduced, even considering the overhead time for the CUDA (Compute Unified Device Architecture) initialization on a widely-used embedded system.

The remainder of this paper is organized as follows. Section 2 describes the optimization problem of the IGC system and MPC with PD-IPM to solve it. Additionally, the real-time problem of PD-IPM is addressed. In Section 3, after computation times for PD-IPM are profiled in a block-wise manner, a new parallelization method for the KKT condition construction part of PD-IPM is proposed. In Section 4, the evaluation results of the proposed method are shown and quantitatively compared with other methods on a widely-used embedded system. Finally, Section 5 presents the conclusions.

## 2. Problem Description

**Figure 1:**
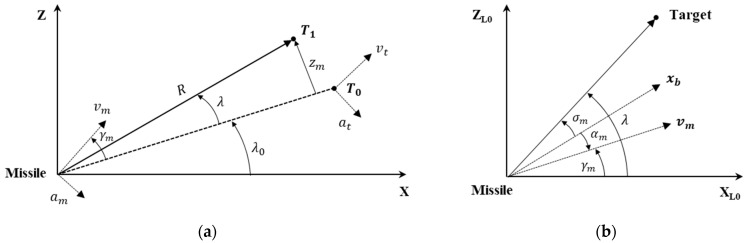
Missile terminal homing phase geometry in a two-dimensional plane (**a**) Planar engagement geometry; (**b**) engagement geometry defined on initial LOS frame.

For the IGC problem, we considered missile terminal homing phase geometry in a two-dimensional plane. Figure 1a depicts planar homing engagement geometry, where the subscripts m and t denote the missile and target. Reference coordinate system X–Z is centered at the missile’s center of gravity; initial target position $T_{0}$ and deviated target position $T_{1}$ are defined on the reference coordinate. Initial line-of-sight (LOS) angle $\lambda_{0}$, LOS angle displacement from initial LOS frame λ, and range-to-go R are also represented. Missile acceleration, velocity, and flight-path angle are denoted by $a_{m},\;v_{m},\;\gamma$, respectively. Relative displacement $z_{m}$ is defined as a normal distance between the target position and initial LOS. In Figure 1b, the seeker look angle $\sigma_{m}$, angle of attack $\alpha_{m}$, and body-fixed coordinate system $x_{b}$ are denoted. Reference coordinate frame X_L0_–Z_L0_ is the initial LOS frame whose origin is also located at the missile’s center of gravity. It is assumed that, in the terminal homing phase, the distance between the missile and target is small enough so that linearization can be performed on the initial LOS frame. Additionally, missile velocity is assumed to be constant. 

The main objective of terminal homing is actuating the missile to intercept the target under the finite maneuver capacity and seeker look-angle limit. In addition, based on the previous study [13], the look-angle rate is limited in bound to prevent image distortion and signal intensity reduction problems. With the acceleration limit $a_{max}$, look-angle limit $\sigma_{max}$, and look-angle rate limit ${\dot{\sigma }}_{max}$, the constraints can be expressed as follows:

(1) $$-a_{max}\leq a_{m}\leq a_{max},-\sigma_{max}\leq \sigma_{m}\leq \sigma_{max},-{\dot{\sigma }}_{max}\leq {\dot{\sigma }}_{m}\leq {\dot{\sigma }}_{max}$$

### 2.1. Augmented Model for Integrated Guidance and Control

Considering the missile short-period dynamics, kinematics, and actuator dynamics, augmented continuous equations for IGC are given by [9,12,13]

(2) $$\underset{\dot{x}}{\underbrace{\left[\begin{array}{c} {\dot{\delta }}_{m}\\ {\dot{\alpha }}_{m}\\ {\dot{q}}_{m}\\ {\dot{\gamma }}_{m}\\ {\dot{z}}_{m} \end{array}\right]}}=\underset{\;\bar{A}}{\underbrace{\left[\begin{array}{ccccc} -\omega_{a} & 0 & 0 & 0 & 0\\ Z_{\delta } & Z_{\alpha } & 1 & 0 & 0\\ M_{\delta } & M_{\alpha } & M_{q} & 0 & 0\\ -Z_{\delta } & -Z_{\alpha } & 0 & 0 & 0\\ 0 & 0 & 0 & \upsilon_{m} & 0 \end{array}\right]}}\underset{x}{\underbrace{\left[\begin{array}{c} \delta_{m}\\ \alpha_{m}\\ q_{m}\\ \gamma_{m}\\ z_{m} \end{array}\right]}}+\underset{\bar{B}}{\underbrace{\left[\begin{array}{c} \omega_{a}\\ 0\\ 0\\ 0\\ 0 \end{array}\right]}}\underset{u}{\underbrace{\delta_{c}}}$$

where $q_{m}$ is pitch rate, $\delta_{c}\;$ is control fin command, and $\delta_{m}$ is actuator response. $Z_{\delta },\;Z_{\alpha },\;M_{\delta },\;M_{\alpha },\;M_{q},\;Z_{\delta }$ and $Z_{\alpha }$ are aerodynamic dimensional derivatives. The actuator dynamics are modeled as a 1st-order lag system with time constant $1/\omega_{a}$.

Equation (2) is characterized by its input and state variables. Compared to conventional guidance and control design, augmented equations for IGC simultaneously consider target-missile kinematics and dynamics. For simplicity, state variable vector and input are represented as x, u. System and input matrices are denoted as $\bar{A}\in R^{5\times 5},\;\bar{B}\in R^{5\times 1}$. Equation (2) is discretized with sampling interval Δt.

(3) $$x_{k+1}=Ax_{k}+Bu_{k}$$

System and input matrices of the discretized equation are $A=e^{\Delta t\bar{A}}$,$\;B=\left(\int_{0}^{\Delta t}e^{\tau \bar{A}}d\tau \right)\bar{B}$, respectively. The notation k represents sampling time step. 

As mentioned above, control input u should be generated within the extent that it does not violate the restrictions. Inequality constraints defined in Equation (1) are linearized and expressed in matrix form [12,13]. As shown below, linearized matrix $C_{k}$ is time-varying. $R_{k}$ is range-to-go in kth time step.

(4) $$\underset{C_{k}}{\underbrace{\left[\begin{array}{ccccc} v_{m}Z_{\delta } & v_{m}Z_{\alpha } & 0 & 0 & 0\\ -v_{m}Z_{\delta } & -v_{m}Z_{\alpha } & 0 & 0 & 0\\ 0 & -1 & 0 & -1 & 1/R_{k}\\ 0 & 1 & 0 & 1 & -1/R_{k}\\ 0 & 0 & -1 & -v_{m}/R_{k} & -\left(v_{m}+v_{t}\right)/R_{k}^{2}\\ 0 & 0 & 0 & v_{m}/R_{k} & \left(v_{m}+v_{t}\right)/R_{k}^{2} \end{array}\right]}}\underset{x}{\underbrace{\left[\begin{array}{c} \delta_{m}\\ \alpha_{m}\\ q_{m}\\ \gamma_{m}\\ z_{m} \end{array}\right]}}\leq \underset{d}{\underbrace{\left[\begin{array}{c} a_{max}\\ a_{max}\\ \sigma_{max}\\ \sigma_{max}\\ {\dot{\sigma }}_{max}\\ {\dot{\sigma }}_{max} \end{array}\right]}}$$

(5) $$R_{k}=R_{0}-k\left(v_{m}+v_{t}\right)\Delta t$$

Overall, the optimization problem for MPC-based IGC is formulated as follows:

(6) $$\mathrm{minimize}\;u_{0},\cdots ,u_{N-1}\;\;\;\sum_{k=0}^{N-1}\left(x_{k}^{T}Q_{k}x_{k}+u_{k}^{T}R_{k}u_{k}\right)+x_{N}^{T}Q_{N}x_{N}\;\mathrm{subject}\;\mathrm{to}\;\;\;x_{k+1}=Ax_{k}+Bu_{k}+b\;C_{k}x_{k}\leq d$$

where $N=\left(t_{f}-t_{0}\right)/\Delta t$ is the size of finite horizon, and $Q_{k}\in {\mathbb{R}}^{5\times 5}$ and $R_{k}\in \mathbb{R}$ are weightings for state variable and input. As all the equations of dynamics and constraints in Equations (3) and (4) are linear, a basic linear MPC framework is adopted. Equation (4) can be transferred into a single-term quadratic problem by introducing new variable $y=\left[u_{0}^{T},\;x_{1}^{T},\;u_{1}^{T},\;x_{2}^{T},\cdots ,\;u_{N-1}^{T},\;x_{N}^{T}\right]$.

(7) $$\mathrm{minimize}\;y\;\;\;y^{T}\Psi y\;\mathrm{subject}\;\mathrm{to}\;\tilde{A}y=\tilde{B},\;\;\tilde{C}y\leq \tilde{D}$$

(8) $$\Psi =\left[\begin{array}{ccccc} R_{0} & & & & \\ & Q_{1} & & & \\ & & \ddots & & \\ & & & R_{N-1} & \\ & & & & Q_{N} \end{array}\right],\tilde{A}=\left[\begin{array}{cccccccc} B & -I & & & & & & \\ & A & B & -I & & & & \\ & & & & \ddots & & & \\ & & & & & A & B & -I \end{array}\right]\;\tilde{C}=\left[\begin{array}{ccccccc} 0 & C_{1} & & & & & \\ & & \ddots & & & & \\ & & & 0 & C_{N-1} & & \\ & & & & & 0 & C_{N} \end{array}\right],\tilde{B}=\left[\begin{array}{c} -Ax_{0}\\ 0\\ \vdots \\ 0 \end{array}\right],\tilde{D}=\left[\begin{array}{c} d\\ d\\ \vdots \\ d \end{array}\right]$$

### 2.2. The Problem of PD-IPM for Real-Time MPC

MPC relies on the real-time solution of a convex optimization problem. The optimization problem in Equation (8) should be defined and computed at every calculation time. To solve the problem, the Primal-Dual Interior Point Method (PD-IPM) was applied, as shown in Algorithm 1. According to the expressions defined in Equations (7) and (8), Algorithm 1 presents a flow chart of PD-IPM for solving a given optimization problem. PD-IPM is one of the most famous algorithm to solve the convex optimization problem. The algorithm alleviates inequality constraints using barrier function and minimize residuals based on perturbed KKT conditions. It attains Newton step computations in every iteration.

In the whole IGC process, the PD-IPM in MPC requires the most computation time because the PD-IPM repeatedly conducts matrix operations until the cost converges. Therefore, the PD-IPM needs to be highly accelerated to be applied to real-time MPC in the IGC process.
**Algorithm 1.** Primal-Dual Interior Point Method [24].**Choose**$\mu \in \left(0,\;1\right),\;\alpha \in \left(0,\;1\right),\;y^{0}>0,\;u^{0}>0,\;v^{0}>0,\;\epsilon >0$ **K: maximum iteration,**$f_{0}\left(y\right)=y^{T}\Psi y$**while**$-f{\left(y^{k}\right)}^{T}u^{k}>\epsilon$
 or  
${\left(\|r_{prim}\|{}_{2}^{2}+\|r_{dual}\|{}_{2}^{2}\right)}^{1/2}>\epsilon$

// Find update direction by solving Newton Step
$\mathrm{compute}\;f\left(y^{k}\right)=\tilde{C}y^{k}-\tilde{D}$
$\mathrm{solve}\;\left[\begin{array}{ccc} \nabla^{2}f_{0}\left(y\right)+\underset{i=1}{\sum }u_{i}\nabla^{2}f_{i}\left(y\right) & \nabla f\left(y\right) & {\tilde{A}}^{T}\\ -diag\left(u\right)\nabla f{\left(y\right)}^{T} & -diag\left(f\left(y\right)\right) & 0\\ \tilde{A} & 0 & 0 \end{array}\right]\left[\begin{array}{c} \Delta y^{k}\\ \Delta u^{k}\\ \Delta \upsilon^{k} \end{array}\right]=-\left[\begin{array}{c} r_{dual}\\ r_{cent}\\ r_{prim} \end{array}\right]$
$\theta =min\left(1,\;min\left(-u_{k}^{i}/\Delta u_{k}^{i}:\Delta u_{k}^{i}<0\right)\right)$

// Backtracking Line Search to find *θ*
$\mathrm{while}\;\|r\left(y^{+},\;u^{+},\;v^{+}\right)\|>\left(1-\alpha \theta \right)\|r\left(y,\;u,\;v\right)\|$

$y^{+}=y^{k}+\theta \Delta y^{k},\;u^{+}=u^{k}+\theta \Delta u^{k},\;v^{+}=v^{k}+\theta \Delta v^{k}$

$\mathrm{compute}\;f^{+}=\tilde{C}y^{+}-\tilde{D},\;r_{dual}^{+},\;r_{prim}^{+},\;r_{cent}^{+}$

θ=αθ
// Primal-Dual Update
$\left(y^{k+1},\;u^{k+1},\;v^{k+1}\right):=\left(y^{k}+\theta \Delta y^{k},\;u^{k}+\theta \Delta u^{k},\;v^{k}+\theta \Delta v^{k}\right)$

## 3. Proposed Method

### 3.1. Overview of the Proposed Method

**Figure 2:**
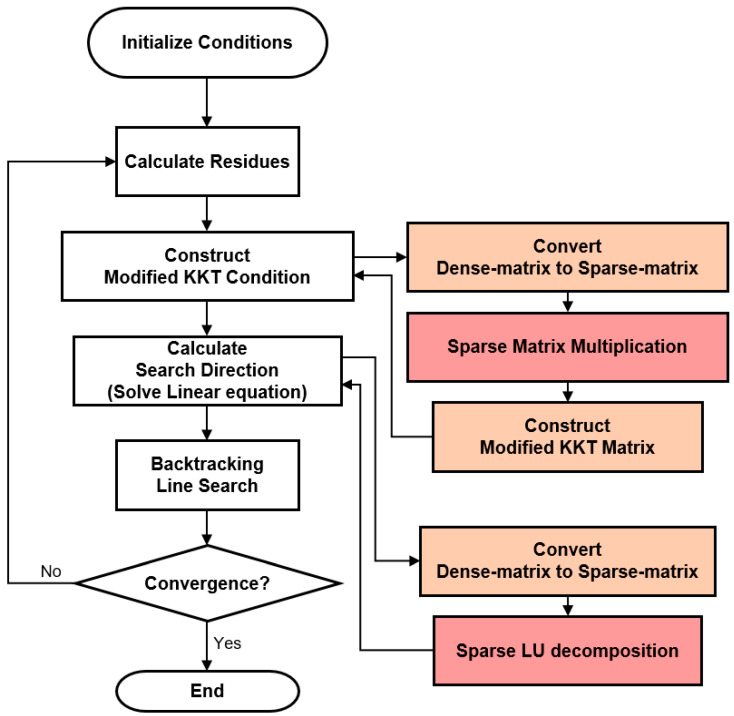
The flowchart of the proposed method.

As shown in Figure 2, the PD-IPM algorithm applied to this application is divided into four parts: (1) calculate residues, (2) construct modified KKT matrix, (3) calculate search direction, and (4) backtracking line search. To improve the computation speed of the algorithm, we measured the computation time for each part of PD-IPM and proposed a method of partially accelerating the parts that required improvements.

### 3.2. Computation-Time Profiling

Computation-time measurements for each part were performed in Nvidia Jetson Xavier NX (20W 6CORE mode), and the results are summarized in Table 2, showing that the construct modified KKT condition and calculate search direction parts take more time than the other two parts. In addition, detailed computation times for these two parts were measured, and the results are shown in Table 3 and Table 4, respectively.

First, Table 3 shows that the sparse matrix multiplication process and construct modified KKT matrix process take a lot of time in the construct modified KKT condition part. Therefore, we set these two parts as parallelization sections and accelerated them to improve the performance. Next, Table 4 shows that the solve linear equation process takes a lot of time in the calculate search direction part. This process is implemented with the SparseLU class in the Eigen library to solve a linear equation using Sparse LU (Lower–Upper) Decomposition. We simply replaced this process with the CUDA cusolver library.

### 3.3. Parallelization Based on CSR and CSC

The modified KKT matrix is obtained through the following matrix operation:

(9) $$S=\frac{1}{2}\left(\left[\begin{array}{cc} 2P-A^{T}B & C^{T}\\ C & 0 \end{array}\right]+{\left[\begin{array}{cc} 2P-A^{T}B & C^{T}\\ C & 0 \end{array}\right]}^{T}\right)$$

where *S* is the modified KKT matrix, *P* is the covariance matrix, *A* is the inequality constraint, *C* is the equality constraint matrix, and *B* is the matrix calculated from the penalty function and equality constraint matrix. To make it easier to compute in parallel, Equation (9) can be simplified as follows.

(10) $$S=\left[\begin{array}{cc} P+P^{T}-\frac{1}{2}\left(A^{T}B\right)-\frac{1}{2}{\left(A^{T}B\right)}^{T} & C^{T}\\ C & 0 \end{array}\right]$$

Then, we divide the top-left sub-matrix of matrix *S* into Equations (11) and (12).

(11) $$D=-\frac{1}{2}\left(A^{T}B\right)$$

(12) $$M=P+P^{T}+D+D^{T}$$

**Figure 3:**
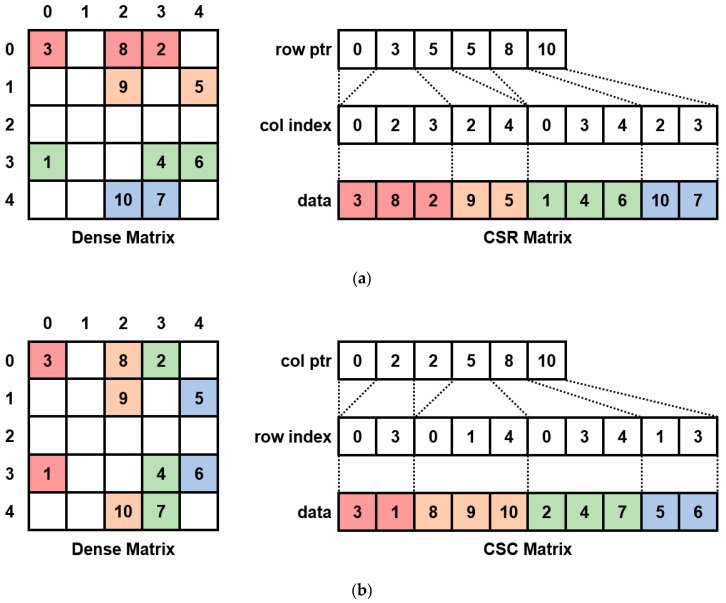
The examples of sparse-matrix conversions: (**a**) Dense to CSR conversion; (**b**) dense to CSC conversion.

Matrices *A* and *B* are large matrices of sizes of about 600 × 600. The multiplication of such a large matrix takes a long time to complete. However, if the matrix contains many zero elements, it can be converted to a sparse matrix to reduce unnecessary operations and the computation time. The Compressed Sparse Row (CSR) and Compressed Sparse Column (CSC) are commonly used formats for sparse matrices, and the conversion exam-ples are shown in Figure 3, respectively. For the CSR format, the accumulated number of non-zero data per row is stored in the ptr array, the column index of non-zero data is stored in the index array, and the element is stored in the data array. As a result, three one-dimensional arrays are created. The CSC format is converted similarly to the CSR format, except that the row changes into a column. As the conversion example shows, the CSR format uses row-wise indexing, whereas the CSC format uses column-wise indexing. Additionally, in matrix multiplication, since the left and right matrices are accessed row-wise and column-wise, respectively, we applied CSR and CSC formats to the left and right matrices, respectively, to increase the matrix access speed. In addition, for a faster operation, sparse matrices *A* and *B* are sorted, and matrix *A* is transposed in advance for the convenience of calculation.

The parallelization algorithm of Equation (11) is shown in Algorithm 2. $A_{d}$ is the non-zero element of matrix *A*, $A_{i}$ is the column index for non-zero elements of matrix *A*, and $A_{p}$ is the cumulative number of non-zero elements for each row of matrix *A*. $B_{d}$ is a non-zero element of matrix *B*, $B_{i}$ is the row index for non-zero elements of matrix *B*, and $B_{p}$ is the cumulative number of non-zero elements for each column of matrix *B*. Additionally, the result matrix *D* is stored as a dense matrix.

The parallel sparse matrix multiplication algorithm shown in Algorithm 2 works as follows. First, a two-dimensional thread is created equal to the size of matrix D, and the algorithm is executed in parallel (Line 1). The non-zero elements of row *r* of matrix A are compared with the non-zero elements of column *c* of matrix B, and multiplication is performed when the column index of matrix A is equal to the row index of matrix B (Lines 7–8). If the row index of matrix B is greater than the column index of matrix A, it means that there is no element with the same index because the matrix is sorted, so the loop is terminated (Lines 5–6). After all the loops are finished, the *sum* variable, in which the multiplication of the (*r*, *c*) element is stored, is calculated to satisfy Equation (11), and finally stored in *D* (Line 12). 

The parallelization algorithm of Equation (12) is shown in Algorithm 3, and the matrix M is calculated using the covariance matrices P and D, which is calculated in the parallel sparse matrix multiplication algorithm. The parallel construct modified KKT matrix (top-left sub-matrix) algorithm shown in Algorithm 3 works by adding elements of matrices P and D, and their transpose matrices in parallel (Line 2).
**Algorithm 2.** Parallel Sparse-Matrix MultiplicationInput* CSR format matrix A(data: $A_{d}$, col index: $A_{i}$, row ptr: $A_{p}$)
* CSC format matrix B(data: $B_{d}$, row index: $B_{i}$, col ptr: $B_{p}$) * Sparse matrices A, B must be sorted.OutputDense matrix D(D)1.all D(r, c) do, in parallel:2.
 *sum*
 ←0
3.  for $i\leftarrow A_{p}\left(r\right)$ to $A_{p}\left(r+1\right)$ do:4.  for $j\leftarrow B_{p}\left(c\right)$ to $B_{p}\left(c+1\right)$ do:5.   if $A_{i}\left(i\right)<B_{i}\left(j\right)$ then:6.    break7.   else if $A_{i}\left(i\right)=B_{i}\left(j\right)$ then:8.
    *sum*
 ← 
*sum*
$+A_{d}\left(i\right)*B_{d}\left(j\right)$
9.end if10.end for11.end for12. D(r,c)←−sum/213.end

**Algorithm 3.** Parallel Construct Modified KKT Matrix (top-left sub-matrix)InputDense matrix P(P), dense matrix D(D)Outputdense matrix M(M)1.all  M(r,c) do in parallel:2.

M(r,c)←P(r,c)+P(c,r)+D(r,c)+D(c,r)

3.end

## 4. Results

### 4.1. Simulation Results

Numerical simulation was performed to compare the computation time. For comparison, terminal homing engagement was assumed. The size of the finite horizon was 1 s, with a sampling time of 0.01 s. Aerodynamic coefficients of missile were set as $Z_{\delta }=-0.2105$, $Z_{\alpha }=-3.1316$/s, $M_{\delta }=160$/s^2^, $M_{\alpha }=-234$/s^2^, $M_{q}=-5$/s. The constant velocities of missile and target were $\upsilon_{m}=380\;$ m/s and $\upsilon_{T}=380\;$ m/s. Fin actuator response was modeled with $\omega_{a}=100$/s. The initial-state variable of the missile was set to $\gamma_{m0}=2\;$ deg, $z_{m0}=20\;$ m. Inequality constraint parameters were $a_{max}=10\;G$, $\sigma_{max}=5\;$ deg, and ${\dot{\sigma }}_{max}=20\;$ deg/s.

**Figure 4:**
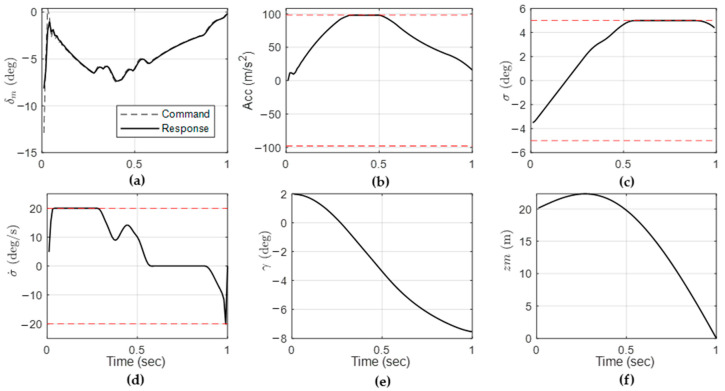
(**a**) actuator command and response; (**b**) acceleration of missile; (**c**) seeker look angle; (**d**) look-angle rate; (**e**) flight path angle; and (**f**) relative displacement between the target and the missile.

Figure 4 shows the single-step simulation results obtained under given design parameters. The optimization problem in Equations (7) and (8) were solved using PD-IPM. The red dotted line on the graph represents the given constraints of acceleration, seeker look angle, and look-angle rate. It is shown that the target was successfully intercepted within the given limitations. 

### 4.2. Results of Algorithm Acceleration with GPU

The test was conducted in Nvidia Jetson Xavier NX (20W 6CORE mode), and parallelization was implemented through CUDA 10.2. We compared the following four implementations: (1) CPU only; (2) CUDA dense: The matrix multiplication section was implemented and parallelized as dense-matrix multiplication; (3) CUDA SpMM: the matrix multiplication section was implemented and parallelized as sparse-matrix multiplication using the csrgemm (CSR × CSR) function in CUDA cusparse library, and (4) ours.

**Figure 5:**
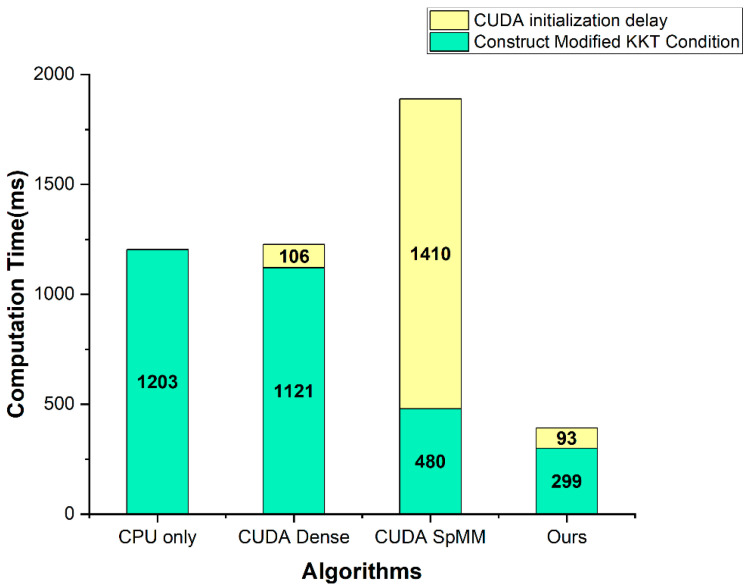
The computation time comparison result graph of the construct modified KKT condition part.

**Figure 6:**
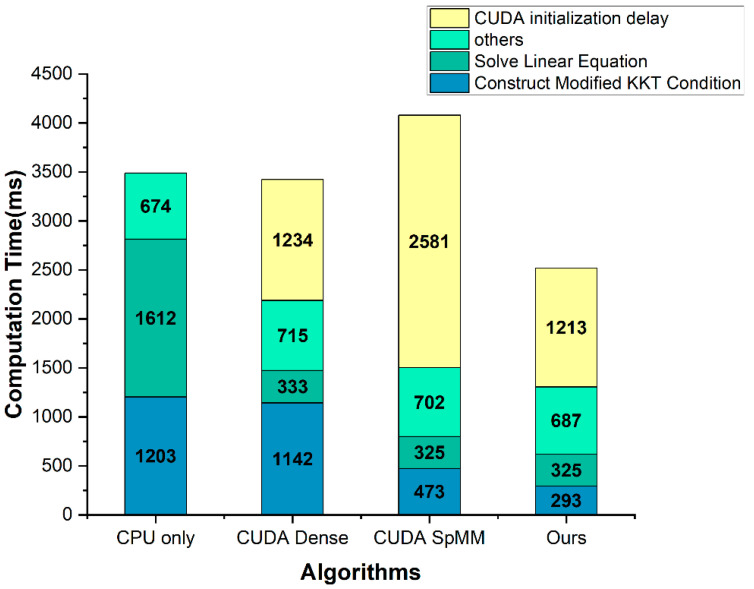
The computation time comparison result graph of the entire application.

Figure 5, Table 5 show the computation time comparison results for the construct modified KKT condition part, and Figure 6, Table 6 show the computation time comparison results for the entire application. In the solve linear equation part, the CPU only was applied with the Eigen library, and the other three methods were improved using the cusolverSpcsrlsvluHost function of the CUDA cusolver library. The CUDA initialization delay is a delay that occurs when calling the CUDA API and initializing the GPU. Therefore, it occurs only once during the entire application runtime and is not directly related to the algorithm.

### 4.3. Analysis

First, Figure 5 and Table 5 show that the CUDA dense and CPU only have almost the same performance. This indicates that parallelizing dense-matrix multiplication makes no sense, since the matrices are very sparse. The CUDA SpMM using the unsorted CSR × CSR multiplication showed about twice the performance compared to the CPU only. However, due to the use of a heavy library, cusparse, there was a long delay problem of the CUDA initialization. On the other hand, our proposed method, Ours, uses CSR × CSC multiplication to improve row and column access speed. Additionally, the CUDA initialization delay is less than the CUDA SpMM because it does not use any additional libraries. As a result, the performance was about 4 times faster than the CPU only, and even including the CUDA initialization delay, the performance was about 3 times faster.

Next, Figure 6 and Table 6 show that the performance of the solve linear equation part in the CUDA dense, CUDA SpMM, and Ours was improved by using the CUDA cusolver library function. However, since the cusolver library is heavy, the CUDA initialization delay increased accordingly. Compared to the CUDA only, the proposed method performed about 2.7 times faster and about 1.4 times faster when the CUDA initialization delay was included.

### 4.4. Discussion 

In this paper, we focused on the parallelization of construct modified KKT condi-tion part in a PD-IPM solver. As shown in Table 2, the construct modified KKT condition part is not the most time-consuming part of the entire computation process. However, it is the part where the efficiency of the parallel operation can be maximized because most of the operations that the construct modified KKT condition part contains are fixed-size sparse-matrix operations.

According to the first result, as shown in Figure 5, our method is more efficient for embedded systems. In general, the CUDA initialization delay does not occupy a large part of the computation time in the desktop environment. However, in an embedded device with a relatively low performance, this delay may take more time than the computation time of the algorithm. In fact, the CUDA SpMM method presented this problem. Our method is suitable not only for desktops, but also for embedded devices because of the short delay. The second result, as shown in Figure 6, indicates that the need for the acceleration of the construct modified KKT condition part as well as the solve linear equation part, and demonstrates that our method works effectively. Because the solve linear equation part takes the most time, other studies have concentrated on that part and improved its performance. However, in an extensive matrix system, such as the IGC, the construct modified KKT condition part also takes a lot of computation time. Therefore, we focused on the acceleration of the construct modified KKT condition part and attained a significant performance improvement.

By adopting CSC and CSR parallelization methods, the computation time of the entire optimization process was significantly reduced. Considering that the CUDA initialization delay occurred just one time, the reduced amount was about 62.5% for the whole flight. This improvement in the context of computation time is highly promising to apply the real-time missile control system. Additionally, since the CUDA used in the proposed method is a widely-used libray for algorithm acceleration, the accomplishment of this paper can be applied to other research areas, such as robotics [37,38,39].

## 5. Conclusions

This paper dealt with the problem of real-time model predictive control (MPC) in integrated guidance and control (IGC) of missile systems. The problem of much computation time in the KKT condition construction part of PD-IPM was firstly addressed and analyzed. A new GPU-based parallelization method was proposed for the KKT condition construction part of PD-IPM. The computation time for PD-IPM was significantly reduced, even considering the overhead time for the CUDA initialization on a widely-used embedded system. The comparison results with the conventional PD-IPM and other methods showed that the proposed method improved the real-time performance by reducing the computation time significantly. In future studies, algorithm acceleration using other hardware, such as FPGA, will be conducted. Additionally, the proposed method will be extended to more algorithms with closed-loop performances, and the stability of a given MPC approach will be evaluated.

## Figures and Tables

**Table 1:** Works related to the acceleration of PD-IPM.

Related works	Target Device	MPC	Parallelization Part	IGC Application
[29]	GPU	○	Linear equation solver	×
[30,31,32,33,34]	GPU	×	Linear equation solver	×
[35]	GPU	×	None	×
[36]	FPGA	○	Linear equation solver	×

**Table 2:** The computation time for each part of PD-IPM.

Part	Computation Time (ms)
Calculate residue	287.285
Construct modified KKT condition	1202.956
Calculate search direction	1612.328
Backtracking line search	352.767

**Table 3:** The computation time for each process of construct modified KKT condition.

Part	Computation Time (ms)
Convert dense matrix to sparse matrix	78.513
Sparse matrix multiplication	695.494
Construct modified KKT matrix	321.575
etc.	108.922

**Table 4:** The computation time for each process of calculate search direction.

Part	Computation Time (ms)
Convert dense matrix to sparse matrix	187.086
Solve linear equation	1425.109

**Table 5:** The computation time comparison results of the construct modified KKT condition part.

Part	Computation Time(ms)			
	CPU Only	CUDA Dense	CUDA SpMM	Ours
CUDA initialization delay	-	106.152	1409.594	92.593
Construct modified KKT condition	1202.956	1120.698	479.706	298.689
Total	1202.956	1226.85	1889.3	391.282

**Table 6:** The computation time comparison results of the entire application.

Part	Computation Time (ms)			
	CPU only	CUDA Dense	CUDA SpMM	Ours
CUDA initialization delay	-	1234.11	2580.534	1213.339
Others	673.505	714.613	701.644	687.447
Solve linear equation	1612.328	332.588	325.038	325.44
Construct modified KKT condition	1202.956	1141.699	473.369	293.119
Total	3488.789	3423.01	4080.585	2519.345
without CUDA initialization delay	3488.789	2188.9	1500.051	1306.006

## Data Availability

Not available.
